# Sphingosine-1-Phosphate Metabolism and Its Role in the Development of Inflammatory Bowel Disease

**DOI:** 10.3390/ijms18040741

**Published:** 2017-03-31

**Authors:** Tomasz Wollny, Marzena Wątek, Bonita Durnaś, Katarzyna Niemirowicz, Ewelina Piktel, Małgorzata Żendzian-Piotrowska, Stanisław Góźdź, Robert Bucki

**Affiliations:** 1 Holy Cross Oncology Center of Kielce, Artwińskiego 3, 25-734 Kielce, Poland; tomwollny@gmail.com (T.W.); marzena.watek@wp.pl (M.W.); stanislaw.gozdz@onkol.kielce.pl (S.G.); 2 Department of Microbiology and Immunology, The Faculty of Health Sciences of the Jan Kochanowski University in Kielce, Aleja IX Wieków Kielc, 25-317 Kielce, Poland; bonita.durnas@onkol.kielce.pl; 3 Department of Microbiological and Nanobiomedical Engineering, Medical University of Białystok, 15-222 Białystok, Poland; katia146@wp.pl (K.N.); ewelina.piktel@wp.pl (E.P.); 4 Department of Hygiene, Epidemiology and Ergonomics, Medical University of Białystok, 15-230 Białystok, Poland; mzpiotrowska@gmail.com

**Keywords:** inflammatory bowel disease, sphingosine-1-phosphate, cancer

## Abstract

Beyond their role as structural molecules, sphingolipids are involved in many important cellular processes including cell proliferation, apoptosis, inflammation, and migration. Altered sphingolipid metabolism is observed in many pathological conditions including gastrointestinal diseases. Inflammatory bowel disease (IBD) represents a state of complex, unpredictable, and destructive inflammation of unknown origin within the gastrointestinal tract. The mechanisms explaining the pathophysiology of IBD involve signal transduction pathways regulating gastro-intestinal system’s immunity. Progressive intestinal tissue destruction observed in chronic inflammation may be associated with an increased risk of colon cancer. Sphingosine-1-phosphate (S1P), a sphingolipid metabolite, functions as a cofactor in inflammatory signaling and becomes a target in the treatment of IBD, which might prevent its conversion to cancer. This paper summarizes new findings indicating the impact of (S1P) on IBD development and IBD-associated carcinogenesis.

## 1. Introduction

Ulcerative colitis (UC) and Crohn’s disease (CD) represent two principal manifestations of inflammatory bowel disease (IBD), a chronic inflammatory disorder of the digestive tract [1]. Both UC and CD present with similar symptoms including diarrhea, abdominal pain, weight loss, and fever [2]. These disorders are characterized by periods of relapse and remission [1,3,4], with destructive chronic inflammation associated with infections, tissue damage, and an increased risk of colon cancer [2]. Many of the mechanisms that regulate mucosal immunity and inflammation are impaired in IBD [5]. Furthermore, evidence suggests that the mechanisms maintaining intestinal epithelial integrity may be reduced, facilitating colonization by pathogenic bacterial strains [2,5,6,7,8,9]. This can alter the systemic immune response leading to the development of fistulas, perforations, infections, abscesses, or dysplasia. Development of these [10] complications correlates with duration of IBD [11,12,13,14]. The current treatment for IBD is aimed at suppression of inflammation in the gut by controlling the abnormal immune response rather than preventing relapse [4]. Recent studies demonstrate that altered leukocyte recruitment in the intestine is a characteristic feature of IBD [5]. Several types of molecules, including sphingosine-1-phosphate (S1P), can regulate leukocyte trafficking to sites of inflammation within the gastrointestinal tract. It is generally accepted that understanding this phenomenon is essential for the development of new treatment methods [2,3]. Moreover, there are the enzymes involved in S1P synthesis or degradation and S1P receptors located in the intestine epithelium, where IBD inflammation arises [15]. On the other hand, the role of S1P in cancer progression was demonstrated by studies showing that activation of sphingosine kinase 1 and production of S1P inhibits apoptosis, allowing survival of cancer cells, and promotes angiogenesis, metastasis, and tumor growth [16].

## 2. Sphingolipids Metabolism

In 1884, using human brain tissue, German biochemist J.L. Thudichum isolated and described the first sphingolipid [17] and gave it the name sphingomyelin. This name recalls the mythical Greek Sphinx creature, to emphasize its enigmatic nature. Recently, the significance of phospholipids and their derivatives in regulating cell function has been appreciated. Initially described for their role as cellular building material or in metabolic processes [10], we now know that lipids can act as signaling mediators responsible for cellular communication [18]. Bioactive lipids include a diverse group of molecules with different chemistries and structures, such as metabolites of arachidonic acid, phosphatidic acid, platelet activating factor, lysophospholipids like lysophosphatidyl choline, and lysophosphatic acid, and certain sphingolipids [19]. Sphingolipid (SL) mediators can be synthesized through de novo pathway or may be derived from the hydrolysis of complex lipids, mainly sphingomyelin (SM), which is present in the plasma membranes of all human cells [20]. Trying to understand SL metabolism and function, one should realize how these molecules are generated, degraded, and where they are located in the cell.

Ceramide (CER) plays a central role in the metabolism of SL. Firstly, it can be synthesized with the condensation of serine and palmitoyl-CoA generating 3-keto-dihydroshingosine, which is then reduced to form sphingamine. Sphingamine is *N*-acelated to produce ceramide (de novo pathway). In the next biosynthetic reactions CER, through the actions of SM synthase, is used to produce sphingomyelin and diacyloglicerol. Secondly, the cleaving of SM by sphingomyelinases may release CER and phosphocholine (hydrolytic pathway). The breakdown of glycosphingolipids catalyzed by specific hydrolases provides another source of CER.

Using the activity of at least three types of ceramidases, which have different subcellular localizations, CER is metabolized to form sphingosine. Sphingosine is then ready for phosphorylation by one of two sphingosine kinases (SPK1 and SPK2) to form S1P.

It is important to understand that SL metabolism reactions take place in different cellular compartments. The first steps of SL de novo pathway (CER formation), are localized on the cytosolic surface of the endoplasmic reticulum (ER) and other ER-like membranes (perinuclear and mitochondria membranes). Synthesis of sphingomyelin occurs in the Golgi apparatus. The transfer of CER from ER to the Golgi apparatus is possible in two different manners. To form SM, CER is derived using ceramide transfer protein. For the synthesis of other SL metabolites (glucosylceramide), CER is derived using the vesicular transport. From the plasma membrane, SL can be recirculated using endosomal pathway. In that way, SM and glucosylceramide are metabolized to CER in the lysosomal compartment. CER is then degraded to form sphingosine and S1P. Since SPH has an ionizable positive charge, it can leave the lysosome and due to adequate solubility in the cytosol, it is able to move between membranes. Therefore, being in the ER, sphingosine is available for recycling and S1P is recognized as a product of turnover. Establishing and understanding of these mechanisms is important to recognize that intercellular localization of SL metabolism pathway is the determinant of bioactive SL site action.

It is interesting that CER, SPH, and S1P cellular concentrations significantly differ with CER presenting the highest, while S1P presents the lowest level. Moreover, a small change in CER could clearly increase the concentration of SPH or S1P.

**Figure 1:**
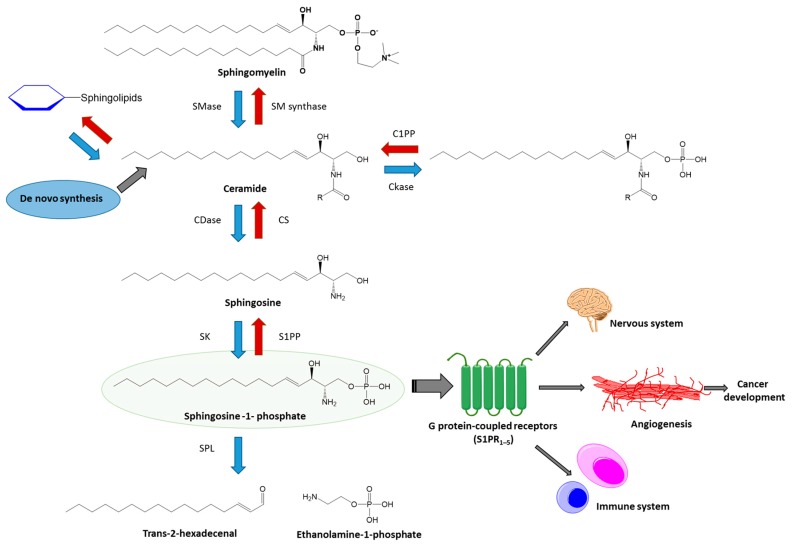
Sphingosine-1-phosphate—its origin and major biological functions. The generation of sphingosine-1-phosphate (S1P) from sphingosine through the hydrolysis of sphingomyelin by sphingomyelinase and then catabolic transformation of ceramide to free fatty acid and sphingosine. Sphingosine might be phosphorylated by sphingosine kinase yielding S1P. S1P can be dephosphorylated back to sphingosine by S1P phosphatase or non-reversibly cleaved by S1P lyase to ethanolamine-1-phosphate and trans-2-hexadecenal. S1P’s biological functions are associated with its ability to activate a family of five G protein-coupled receptors, S1P receptors 1–5 (S1PR1–5). S1P exerts some actions for gastrointestinal tract (GI), nervous system, immune response, and angiogenesis, which is linked with cancer development. Abbreviations: SMase—Sphingomyelin phosphodiesterase; SM synthase—Sphingomyelin synthase; C1PP—ceramide-1-phosphate phosphatase; CKase—ceramide kinase; CDase—ceramidases; CS—ceramide synthase; S1PP—S1P-phosphatase; SK—sphingosine kinase; SPL—S1P lyase; S1PR1–5—five G protein-coupled receptors, S1P receptors 1–5.

The most important lipids of the sphingolipid signaling cascade are presented in Figure 1, including ceramide (CER), ceramid-1-phosphase (C1P), sphingosine (SPH), and sphingosine-1-phosphate (S1P).

Sphingolipids, and especially S1P, were first described as mediators of embryonic development, primarily cardiogenesis [21] and vasculogenesis [22], as well as in development of cancer and metastasis [23]. Currently, attention is focused on their role in cardiovascular and neurological disorders, tumor biology, inflammation, and lymphocyte trafficking [19,24]. Another issue recently underlined is the role of SL and its metabolites in the regulation of cell proliferation and apoptosis [25,26,27]. In this regard, CER and SPH are known to inhibit proliferation and promote apoptosis while S1P-mediated signals are associated with cell growth arrest and apoptosis inhibition [27,28,29,30]. Because SL metabolites are interconvertible, their relative tissue levels play a more important role in the final phenotype than the absolute amount measured [31]. Considering the role of SL in regulating cell growth and apoptosis, it is unsurprising that SL metabolism is altered in cancer, where enhanced cell growth, diminished cell death, or both are well documented [25,26].

## 3. S1P

**Figure 2:**
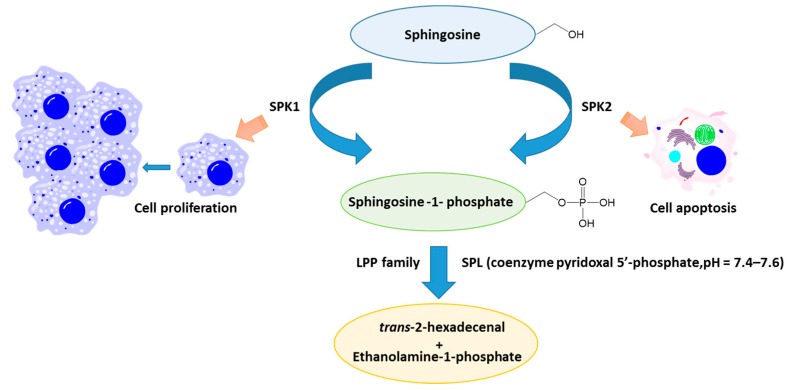
Involvement of S1P in cell apoptosis and cell proliferation. Divergent cellular effect via sphingosine kinase isoform activation. Increasing of cellular proliferation and migration by growth factors and cytokines depend on SPK1 activation and promotion of apoptosis by activation of SPK2. Elimination of S1P via different ways: dephosphorylation reactions catalyzed by S1P-specific phosphatases that require the coenzyme pyridoxal 5′-phosphate and a specific pH range (7.4–7.6) or by enzymes belonging to the nonspecific lipid phosphate phosphatase family (LPPs) which are important in controlling local S1P levels within specific tissue niches. Abbreviations: SPK1—sphingosine kinase 1; SPK2—sphingosine kinase 2; SPL—S1P lyase; LPPs—lipid phosphate phosphatase.

Sphingosine-1-phosphate (S1P) is a bioactive signaling sphingolipid, found in the circulation and most tissues [5]. It is derived from the recycling of endogenous human sphingolipids and the metabolism of dietary animal products containing sphingolipids [32]. S1P activates five different G protein-coupled receptors (S1PR1-5). T cells express G protein-coupled receptors that bind S1P and their circulation depends on S1P gradients. Some of the S1P-mediated effects are not due to S1PR activation (Figure 1). One example is the role of S1P in the IL-6/STAT3 pathway (interleukin 6/signal transducer and activator of transcriptional factor STAT3), which is involved in IBD pathophysiology and colon cancer development [33,34,35,36,37,38,39,40]. Indeed, S1P production is increased in colon cancer [41,42], and S1P is required for TNF alpha (TNFα)-dependent nuclear factor-kappa B activation (NF-κB) [43,44,45]. Moreover, most of the nuclear functions of S1P are not connected to S1PR. S1P is enzymatically released from sphingosine by sphingosine kinase 1 (SPK1) and the tissue-restricted isoform, sphingosine kinase 2 (SPK2). SPK1 was first found in lungs and spleen, while SPK2 was known to be predominantly found in the heart, brain, and liver [46,47,48]. SPK2 activation can promote apoptosis [49], while SPK1 activation by growth factors and cytokines (including TNFα) can lead to cellular proliferation and migration [50]. Alternatively, S1P may be degraded via dephosphorylation by specific and non-specific lipid phosphatases [51] (Figure 2). S1P released from platelets and mast cells promotes wound healing, participates in the inflammatory response [52], liver fibrosis [53], and has different functions in angiogenesis, and innate and adaptive immunity [24]. Sphingosine phosphate lyase (SPL), which irreversibly degrades S1P, is expressed in differentiated enterocytes of both intestines, Paneth cells, and inflammatory cells [51,54]. Moreover, SPL is downregulated in colon cancer which results in increased S1P in neoplastic intestinal tissues, indicating the potential role of S1P/SL in colon carcinogenesis.

## 4. Autoimmune Disease and S1P

The influence of the sphingosine pathway on lymphocyte trafficking is well described. It has been shown that S1PR and SL regulate the exit of lymphocytes from the thymus into the circulatory system [55,56]. Furthermore, the pharmacological inhibition of SL or manipulation of signaling through S1PR induced lymphopenia in mice [57]. This relationship has also been strengthened by genetic studies [58,59]. Modulation of S1PR on lymphocytes in the lymph nodes prevents their flow into the central nervous system, where they are responsible for pathological damage, which is relevant in multiple sclerosis (MS) therapy [60,61,62]. In fact, fingolimod, the first oral S1PR modulator, demonstrated beneficial effects in MS patients both in prophylactic and therapeutic setting, confirming the clinically important ability of S1P to modulate immune response [63,64,65].

The oncogenic effect of sphingosine kinase (SK), responsible for S1P production, was first postulated in the work of Pu Xia et al. [66]. Later, these authors demonstrated the role for SPK in signal transduction leading to NF-κB activation and inhibition of apoptosis [67]. Recent work also has linked S1P signaling not only to autoimmune disease, but also to inflammation and cancer [34]. They showed that S1P-S1PR and STAT3 signaling pathways are able to modulate one another, promoting not only inflammation, but also carcinogenesis. Moreover, in animal models of encephalomyelitis and atherosclerosis S1PR ligands directly activate the STAT3 pathway [68] and S1PR2 activation increased NF-κB activity in macrophages [69]. These observations clearly indicate the important role of S1P in the development of autoimmune diseases and support the study of the role of S1P in other inflammatory disorders of autoimmune origin.

## 5. Inflammation in IBD and S1P

### 5.1. Role of SPK/S1P in Inflammation

S1P converted from sphingosine mainly by SPK1, is released by platelets during inflammation, reaching 100 to 400 nM in serum [70]. When its concentration in the blood is much higher than in the tissue, lymphocytes, due to altering S1P receptor (1 S1PR1) expression, exit from lymphoid organs to circulation. On the other hand, S1PR1 downregulation during peripheral lymphocyte activation correlates with T cells retention in lymphoid tissue [71].

Moreover, there is also an important role for S1P in inflammation development. Binding of S1P to cell-surface G-protein-coupled receptors can activate NF-κB, which is needed for inflammatory and immune responses. It was also shown that S1P resembles the biological effect of TNFα in activating endothelial cells, which occurs through activation of SPK1. There are data showing that SPK1 is the necessary mediator in LPS, TNFα and interleukin beta signaling, and pro-inflammatory functions [16].

### 5.2. In Vitro Experiments

Despite the unknown pathogenesis of IBD, dysregulation of the immune mechanisms that maintain the balance between intestinal mucosa and the gut environment may be important in understanding its origin [2]. Data based on genetic analyses indicate that antimicrobial peptides, autophagy, endoplasmic reticulum stress, innate and adaptive immune cell function, T-helper 17 and regulatory T-cells, TNFα, as well as many interleukins, are important factors in IBD [72,73,74,75]. Moreover, these substances stimulate many pathways that activate crucial inflammatory transcription factors, such as NF-κB and signal transducer and activator of transcription 3 (STAT3), which regulates signals from different gut stimuli [33,76]. Understanding the interactions and cross-talk between intestinal cells, secreted mediators, and transcription factors and whether they are affected during inflammation are of potential importance for the development of new IBD therapy.

The elevated intestinal expression of some genes involved in sphingolipid metabolism was recently reported, prompting exploration of S1P in signaling and subsequent targeting in IBD [77,78]. The authors have studied 13 gene loci that are linked to ulcerative colitis; they found that polymorphism of orosomucoid (ORM) 1-like 3, which is a homologue of the SL regulatory protein ORM1, was associated with UC. The ORM region was selected for analysis since it has been implicated not only in UC, but also in diseases involving dysregulated immune response, including Crohn’s disease and asthma. The genetic associations shown in this work underline the importance of alterations in barrier functions and cell specific innate responses (microbe responses, production of reactive oxygen species, and activation of TNFα). Genes, which play a role in these processes, can regulate important functional programs in adaptive immunity and resolution of inflammation in the pathogenesis of ulcerative colitis.

### 5.3. In Vivo Experiments

TNFα plays a major role in IBD, and its expression is upregulated in the intestine of patients with active disease [79,80]. Furthermore, TNFα can activate SPK1, leading to several downstream effects including increased cyclooxygenase 2 (COX2) and prostaglandin E (PGE2) production, monocyte degranulation, and transcription of adhesion molecules [81,82,83]. The net effect of these responses is severe inflammation. To help elucidate the role of SPK1/S1P in IBD, its expression was examined in patients with active disease and found to be elevated in both colonic epithelial cells and inflammatory infiltrate. Additionally, upregulation of SPK1/S1P pathway correlates with COX2 overexpression [84]. Animal models, particularly colitis, have been important in further defining the role of SPK1/S1P in IBD [84]. These studies showed that SPK1 knock-out mice (SPK1^−/−^) were protected from the development of weight loss, splenomegaly, anemia, and leukocytosis in a dextran sulfate sodium (DSS)-induced colitis model. In addition, blood S1P levels were elevated in wild type (WT) mice, but not in SPK1^−/−^, in response to DSS treatment. Moreover, increased inflammatory infiltrate, SPK1 activity, and COX2 expression were observed only in wild type mice, while SPK^−/−^ mice were less susceptible to DSS colitis. Mechanistically, the authors suggest that the increased S1P in IBD is downstream of TNFα and upstream of COX2. Collectively, these data strongly indicate that the SPK1/S1P pathway is involved in the development of inflammation and persistence of IBD. It is well established that neutrophil infiltration into the crypts and lamina propria of the colon is a major feature in IBD. Additionally, S1P promotes leukocyte migration. The observation that only the SPK1^−/−^ mice showed a failure of granulocytic infiltration in colon in the DSS model along with the observation that colonic SPK1 activity and the S1P concentration were significantly elevated only in SPK1 null mice suggests that S1P may function as a chemoattractant for granulocytes in the colons of WT mice [84]. Therefore, downregulation of S1P by inhibition of SPK1 may be an important therapeutic target for controlling systemic and local inflammation in IBD [84].

New data providing evidence for the role of S1P in inflammatory cell traffic to affected intestine in ulcerative colitis was published recently [85]. Sandborn et al studied the effect of orally administered S1P receptor 1 and 5 agonist (ozanimod) in UC patients. This small molecule drug, when bound to S1PR1 and 5, leads to their functional antagonism by internalization and degradation of the receptors; in that way the lymphocytes remain trapped in lymph nodes and clinical remission of UC is expected. Therefore, a randomized, double blind phase II clinical trial in patients with UC was conducted: patients received ozanimod at the dose of 0.5 or 1.0 mg or placebo orally, as induction and 32 weeks therapy. Clinical remission was achieved at week 8 in 16% of patients on ozanimod 1 mg, in comparison to 6% patients receiving placebo, being statistically significant (however, it was not for the group on azanimod 0.5 mg). Also patients treated with 1 mg of ozanimod had significantly better secondary outcomes, including clinical response at week 8 (placebo-7%, 0.5 mg ozanimod-54%, *p* = 0.06, 1 mg ozanimod-57%, *p* = 0.02), mucosal healing at week 8 (placebo-12%, 0.5 mg ozanimod-28%, *p* = 0.03, 1 mg ozanimod-34%, *p* = 0.002), as well as histologic remission (placebo-11%, 0.5 mg ozanimod-14%, *p* = 0.63, 1 mg ozanimod-22%, *p* = 0.07). The authors reported that ozanimod had a safer profile; only few adverse effects were noted. Sandborn demonstrated that ozanimod not only induced symptoms resolution, but also endoscopic healing and histologic absence of inflammation in UC patients. However, whether this drug will find a role in future IBD treatment, phase III trials should clearly reveal.

## 6. Cancer Associated with IBD

**Figure 3:**
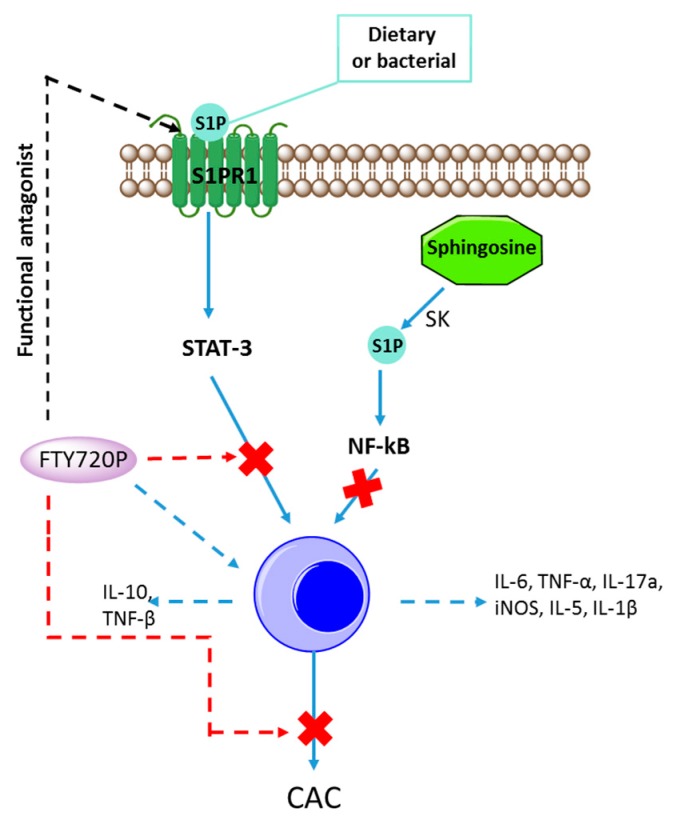
Immunomodulatory activity of FTY720P. FTY720P as functional antagonist indirectly diminishes STAT-3 signaling by binding to S1PR1 on the cell surface, resulting in the internalization of S1PR1, and preventing S1P from binding to and activating this receptor. Binding of FTY720P to the receptor thus causes the receptor to be sequestered inside the cell, out of reach of S1P. Solid arrows indicate S1P-dependent signaling pathways; dashed arrows indicate the impact of functional antagonist—FTYY720P on signaling pathways. Abbreviations: S1P—sphingosine-1-phosphate; S1PR1—sphingosine-1-phosphate receptor1; FTY720P—fingolimod phosphate (*S*)-enantiomer; SK—sphingosine kinase; STAT3—signal transducer and activator of transcription 3; NFκB—nuclear factor kappa-light-chain-enhancer of activated B cells; CAC—colitis and cancer; red cross means that this pathway is inhibited.

A growing body of evidence supports the observation that chronic inflammation in the colon is a key factor leading to malignant tumor development. Indeed, ulcerative colitis markedly increases the risk of colorectal cancer [43,86]. In animal models, the association between colitis and cancer (CAC) is linked to NF-κB and STAT3 pathways [72,87,88], which are known to stimulate malignant cell growth and tumor formation. Moreover, STAT3 and NF-κB are both responsible for promoting inflammation by increasing the expression of well-known proinflammatory cytokines such as TNFα and IL-6, which in turn lead to cancer initiation and progression [88,89]. These observations have been confirmed in epidemiological studies, where an association between the prevalence of colorectal adenomas and increased levels of IL-6 and TNFα are shown [90]. There is evidence that S1P as well as SPK1 and SPK2 are involved in mediating the effects of proinflammatory cytokines such as TNFα [82,91]. Moreover, TNFα activates and governs SPK1 translocation to the plasma membrane, where it is responsible for S1P formation [92]. Previous studies have demonstrated that S1P plays an important role not only in inflammatory processes, but also in cancer development [45,93,94,95]. Moreover, the S1P receptor (S1PR1) was found to be responsible for persistent STAT3 activation in gastric tumors and in diffuse large B-cell lymphomas [34,96]. These authors documented that S1PR1 expression was induced by STAT3 and, conversely, that persistent STAT3 activation in tumors was dependent on the presence of S1PR1 in malignant tissue and associated immune cells [34]. In fact, S1P may activate the production of the NF-κB-regulated cytokine, IL6, which is involved in the pathogenesis of both IBD and CAC [45,97,98]. Using SPK2 knockout mice in a model of CAC colitis, Liang and coworkers [57] demonstrated an intriguing association between SPK1 and SPK2 activity. They showed that SPK2 knockout mice had increased circulating and colonic S1P levels when compared to controls. Trying to elucidate this paradox, they suggested that reduction of nuclear SPK2 activity in knockout mice could upregulate SPK1, leading to an increase in S1P. Moreover, they found that tumor number and size were higher in SPK2 knockout mice versus wild type mice [57]. It was found that NF-κB activation and IL-6 and S1PR1 expression were all significantly increased in SPK2 knockout mice colons when compared to controls. To explain the role of S1P in CAC, it has been proposed that an SK/S1P/S1PR1 axis could activate NF-κB and mediate continuous STAT3 activation (thus leading to the expression of STAT3-dependent gene products, such as c-Myc), resulting in CAC (Figure 3). In an effort to confirm this hypothesis, an S1PR1 functional antagonist FTY720 was administered. Treatment with FTY720 reduced the STAT3 cascade and S1PR1 activation, which prevented CAC in SPK2 knockout mice. Since FTY720 also reduced colitis activity, it should be explored as a potential drug in IBD patients. However, association of FTY720P dependent disruption of S1P/SPK1/S1PR1 signaling loop and its clinical importance in IBD require further studies [64,99].

## 7. Sphingolipids in Diet

An average of 0.3–0.4 g sphingolipids, which are metabolized to ceramides, are consumed every day [100]. In animal models of IBD and cancer, the role of sphingolipids as dietary active molecules has been extensively studied [101,102,103]. It was demonstrated that in an inflammation and oxidative stress-dependent model of colon cancer induced by 1,2 dimethylhydrazine (DMH), dietary sphingomyelin is able to reduce premalignant lesions and tumor development by 20% [104]. This effect might be explained by the decreased expression of transcription factors; hypoxia-inducible factor 1α and transcription factor 4 that are important in tumorigenesis, and are activated following sphingolipid treatment [103]. These observations are in accordance with previously published data [105] exploring the role of dietary sphingomyelin in a mouse model of CAC. The addition of 1 g/kg of dietary sphingomyelin significantly decreased the disease activity index and tumor number in mice. The authors suggest that sphingomyelin changed the expression of some pro-inflammatory cytokines (interferon gamma, IL-17, IL-23) and increased anti-inflammatory ones (IL-4, IL-3, IL-13ra2, IL-10rb) in their experimental model. However, other findings showed that sphingomyelin can aggravate mucosal inflammation by increasing apoptosis of colon epithelial cells in an animal model of colitis [106]. Therefore, the authors suggested that precise sphingomyelin doses, its microscopic structure, and the state of targeted tissue could decide if dietary sphingolipids were beneficial or harmful in inflammation and long-term chemoprevention of CAC.

Another explanation of this phenomenon was raised in the form of a hypothesis in a recently published work [107]. These authors suggested that sphingolipids derived from mammalian food sources contain a backbone that could be converted to S1P in gut epithelium. In the inflamed IBD colon, S1P would be able to impact on STAT3 signaling, cytokine induction, and CAC. On the other hand, dietary sphingolipids containing a different structural back bone would not be converted to S1P and could not promote inflammation and CAC. This was supported by the observation that sphingadienes, a dietary metabolites derived from sphingolipids of soy, reduced colon tissue S1P levels, STAT3 activation, and cut CAC in animal models [107].

Therefore, it seems that dietary sphingolipids may represent a simple strategy for reducing IBD colitis and preventing colon carcinogenesis.

## 8. Conclusions

Currently, our understanding of the development and pathophysiology of IBD is limited. Based on recent data, it is possible that sphingolipids, in particular the SPK/S1P signaling and metabolism, represent a missing link in the understanding the IBD causes and effective therapy. S1P is an active signaling mediator that may have a profound impact on both IBD development and progression. Moreover, S1P/S1PR1 signaling pathways in animal models of colitis are responsible for CAC development. FTY720, an S1PR1 functional antagonist, could prevent not only inflammatory progression, but also tumor formation in animal models of colitis. Orally administered S1P receptor 1 and 5 agonist, ozanimod resulted in significant benefit over placebo in UC patients.

We believe that further efforts to understand how sphingolipids could influence the homeostasis of the immune functions of the gut are needed. Modulation of sphingolipids signaling might help to diminish the extent and severity of inflammation in IBD and prevent cancer development in patients at risk.
